# The Role of Infill Density in Impact Localization for Additively Manufactured Structures

**DOI:** 10.3390/s26092720

**Published:** 2026-04-28

**Authors:** Hussain Altammar

**Affiliations:** Department of Mechanical Engineering, College of Engineering, King Faisal University, Al-Ahsa 31982, Saudi Arabia; haltammar@kfu.edu.sa

**Keywords:** additive manufacturing, infill density, fused deposition modeling, impact localization, group velocity, genetic algorithm

## Abstract

The optimization of impact localization in 3D-printed structures is critical for their application in smart monitoring and damage detection systems. This study investigates the influence of infill density on the accuracy of low-velocity impact localization in 3D-printed plates. Specimens with cubic infill patterns and varying densities (30%, 50%, and 100%) were fabricated and subjected to impacts with varying locations and magnitudes using two different sensor network configurations. A genetic algorithm integrated with continuous wavelet transform was employed to simultaneously determine impact coordinates and group velocity. Key findings reveal that lower infill structures act as mechanical low-pass filters, producing clean and low-frequency signals, while higher densities support complex wave propagation with higher energy and broader frequency content. The dominant frequency of first arrival shifts toward lower values with increasing impact energy across all densities. Group velocity increases with both impact energy and infill density. For 30% infill, it averages around 450 m/s, while for 100% infill it exceeds 800 m/s. The genetic algorithm demonstrated robust performance across all experimental conditions, simultaneously estimating impact coordinates and group velocity with average errors below 6% for all infill densities. Spatial probability mass functions revealed tightly clustered predictions around true impact locations, with maximum probabilities reaching 68% and uncertainties below 5%. Computational efficiency varied modestly with infill density. These findings provide quantitative relationships between infill density, wave propagation characteristics, and localization performance for designing a reliable structural health monitoring of additively manufactured structures.

## 1. **Introduction**

Additive manufacturing (AM) has emerged as a transformative paradigm in modern engineering, offering design freedom, material efficiency, and geometric complexity that conventional manufacturing methods cannot achieve [1,2]. These unique capabilities have generated immense interest across the aerospace, mechanical, civil, and biomedical engineering sectors, where AM components are increasingly deployed in load-bearing applications, unmanned aerial vehicles, and infrastructure monitoring systems [3,4,5,6]. A particularly promising direction lies in the seamless integration of AM with embedded or surface-mounted sensors during fabrication, creating intelligent structures that are capable of self-sensing and real-time health monitoring [7,8]. This convergence enables direct embedding of fiber Bragg gratings, piezoelectric elements, and conductive pathways within printed components, eliminating post-processing assembly and reducing system complexity [9,10,11].

While structural health monitoring (SHM) methodologies are well established for metals and composites, their application to AM structures introduces new challenges. Fused deposition modeling (FDM), a widely used AM technique for thermoplastics such as polylactic acid (PLA), produces components with internal mesostructures that fundamentally alter stiffness, anisotropy, and wave propagation behavior. Extensive mechanical studies have characterized how printing parameters influence quasi-static properties. Higher infill densities and optimized patterns consistently increase stiffness and strength across tensile, bending, and impact loading conditions [12,13,14,15]. Printing parameters such as temperature, speed, and wall perimeter strongly influence elastic properties, with full-field characterization revealing up to 20% variation in modulus, depending on infill architecture [16,17,18]. Carbon-nanofiber-modified PLA exhibits piezoresistive behavior, enabling strain sensing directly within printed components [19]. Embedded fiber Bragg grating sensors show that the infill pattern and density significantly affect strain transfer and metrological accuracy [9]. Hybrid SHM approaches reveal that lower-density PLA plates experience stronger wave attenuation under repeated impacts, whereas fully dense plates exhibit improved detectability [20]. Embedded piezoelectric transducers have also demonstrated reliable real-time SHM across varying infill patterns and densities [10].

Despite this comprehensive understanding of quasi-static mechanical behavior, a critical gap remains. The influence of infill density on guided wave propagation and impact localization accuracy has not been systematically investigated. This gap is particularly significant, as AM structures increasingly appear in applications where real-time impact detection is essential for structural integrity [21,22].

Impact localization in plate structures typically relies on time-of-arrival (ToA) or time-difference-of-arrival (TDoA) methods [23]. Lamb wave studies have shown that low-energy impacts in composite plates can be detected using selectively excited modes and time-of-flight estimation, even with sparse piezoceramic networks [24,25]. TDoA-based multilateration can locate impacts in isotropic plates without prior knowledge of wave velocity, making it robust against temperature- and frequency-dependent variations [26]. Hybrid ToA/TDoA frameworks further improve accuracy by optimizing sensor–target geometry and employing iterative solvers such as Gauss–Newton [27]. Recent advances emphasize probabilistic and uncertainty quantification approaches. Probabilistic group-velocity mapping combined with simulated annealing has demonstrated high-confidence localization without baseline data [28]. Related probabilistic frameworks incorporate environmental variability to identify defects through multi-level probability mass functions [29]. Broader uncertainty-aware damage identification approaches reduce false positives and improve robustness under system disturbances and measurement noise [30].

Wave-based impact localization increasingly relies on advanced signal processing and optimization techniques to address challenges posed by dispersive elastic waves and noisy measurements [31,32,33,34,35]. Genetic algorithms (GAs) have proven effective for feature extraction, parameter estimation, and damage localization in SHM applications [36,37]. However, these challenges become more pronounced in AM plates, where the infill pattern and density actively shape wave propagation, complicating the localization of low-velocity impacts that often produce barely visible or internal damage [38,39].

This study is the first systematic investigation considering infill density as a governing parameter for impact localization in additively manufactured structures. This paper addresses this gap by investigating the influence of infill density variations (30%, 50%, and 100%) on impact localization in FDM-printed PLA plates with integrated piezoelectric sensors. Using a genetic algorithm-based computational framework as a data processing tool, we evaluate the effects of infill density on wave propagation characteristics, localization accuracy at multiple test points, and computational cost.

The remainder of this paper is structured as follows: Section 2 details the methodology, including specimen fabrication, experimental setup, sensor configurations, and the GA formulation. Section 3 presents the results, providing comprehensive evaluation of localization accuracy and estimated wave velocities across different infill densities. Section 4 offers concluding remarks.

## 2. Methods

### 2.1. Specimen Design and Fabrication

**Figure 1:**
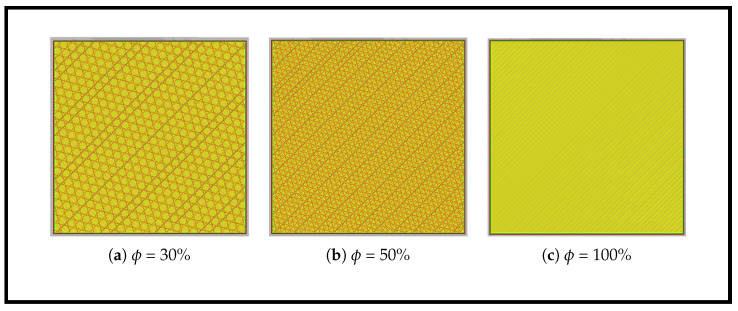
Schematic of the cubic infill patterns with various infill densities captured at the 5th layer.

The experimental investigation employed PLA plates fabricated using a fused deposition modeling (FDM) printer. To systematically evaluate the effects of internal architecture on wave propagation and localization accuracy, three distinct infill densities were manufactured: 30%, 50%, and 100%. A cubic infill pattern was selected for its mechanical behavior and structural stability, ensuring that variations in dynamic response could be attributed primarily to changes in material volume fraction rather than directional stiffness effects. Figure 1 illustrates the progression from 30% to 100% infill, revealing a clear transition from a sparse, porous internal architecture to a nearly solid structure. This provides a controlled basis for examining the influence of mesostructure on wave propagation. The PLA filament used for fabrication has material properties of $\rho \approx 1240\;\mathrm{kg}/m^{3},E\approx 3.16\;\mathrm{GPa},$ and ν≈0.33.

**Figure 2:**
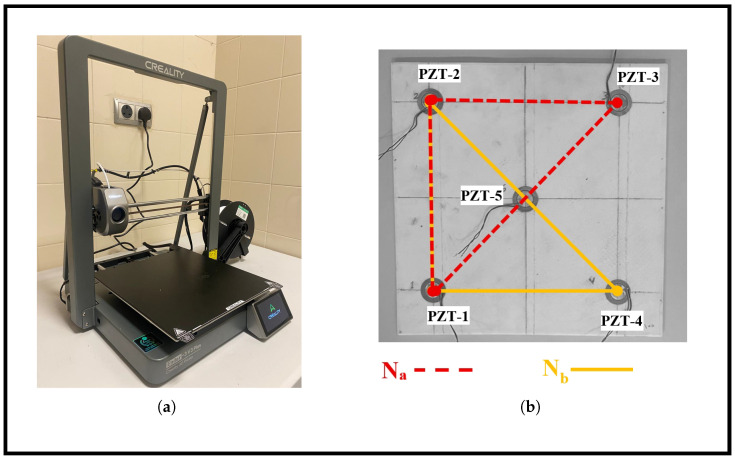
(**a**) Creality Ender 3 V3 Plus FDM printer with build volume of 300 × 300 × 330 mm^3^; (**b**) AM plate with five piezoelectric sensors showing two sensing networks.

All specimens were printed using consistent manufacturing parameters to isolate the effect of infill density. A Creality Ender 3 V3 Plus (Shenzhen Creality 3D Technology Co., Ltd., Shenzhen, Guangdong, China), shown in Figure 2a, was used for all fabrications. All plates were printed flat on the build plate with the same orientation. The printing parameters included a nozzle diameter of 0.4 mm, a nozzle temperature of 215 °C, a heated bed temperature of 65 °C, and a printing speed of 88 mm/s. Additional settings were maintained uniformly across all plates, including a layer height of 0.2 mm, strut thickness of 0.4 mm, a wall thickness of 0.8 mm, and structural orientation of $\pm 45^{\circ }$. These parameters ensured stable extrusion, repeatable layer bonding, and consistent geometric fidelity across all specimens. No post-processing treatments were applied to the printed plates, so as to preserve the as-printed surface conditions and internal structure.

To capture the waveform signals generated by low-velocity impacts, circular PZT piezoelectric sensors were bonded to the surface of each AM plate. The sensors exhibited material properties of $\rho \approx 7800\;\mathrm{kg}/m^{3},E\approx 0.5\;\mathrm{GPa},$ and a piezoelectric strain coefficient of $d_{31}=-276\times 10^{-12}\;C/N$ [40]. Each sensor had a diameter of 11 mm and a thickness of 0.4 mm. These dimensions were selected to ensure high sensitivity while minimizing added mass and stiffness effects on the structure [41].

Five sensors, denoted as $S_{1}$ through $S_{5}$, were positioned at predefined coordinates to form sensing networks. As illustrated in Figure 2b, the five sensors defined two distinct triangular sensing networks used for comparative analysis of wave propagation and localization performance. Network $N_{a}$ comprised sensors $S_{1}$, $S_{2}$, $S_{3}$, and $S_{5}$, forming a triangle that spans the upper region of the plate. Network $N_{b}$ included $S_{1}$, $S_{2}$, $S_{4}$, and $S_{5}$, forming a complementary triangle across the lower region. These configurations allowed us to assess how sensor geometry influences the accuracy of impact localization under varying infill conditions. The sensors coordinates were as follows: $S_{1}$ at (50, 50) mm, $S_{2}$ at (50, 250) mm, $S_{3}$ at (250, 250) mm, $S_{4}$ at (250, 50) mm, and $S_{5}$ at the center (150, 150) mm.

Table 1 summarizes the measured physical and geometric properties of the fabricated plates. A clear monotonic increase in mass can be observed as the infill density increases, rising from 270±2.6 g for the 30% infill plate to 558±1.3 g for the fully dense 100% specimen. This trend is accompanied by a corresponding increase in effective density, which progresses from 0.589 g/cm^3^ at 30% infill to 1.190 g/cm^3^ at 100% infill. The intermediate 50% plate exhibits mass and density values of 369±1.5 g and 0.780 g/cm^3^, respectively.

The geometric dimensions in Table 1 show minor variations. The measured thickness increased slightly with infill density, from 5.3±0.11 mm (30%) to 5.5±0.09 mm (100%), while the in-plane dimensions remained highly consistent across all plates, with widths and lengths ranging from approximately 292 to 296 mm and standard deviations below ±1 mm. The calculated volumes (458.11–473.13 cm^3^) show similarly small variation, falling well within the expected tolerance range for the FDM printing. These results establish a quantitative baseline for interpreting the dynamic behavior of the specimens and impact localization accuracy under varying infill densities.

### 2.2. Impact Generation and Data Acquisition

Low-velocity impacts generate elastic waves that propagate radially from the contact point through the plate structure. In thin plates, these waves manifest primarily as fundamental guided wave modes, whose propagation characteristics are governed by the material properties and geometric constraints of the medium. For AM plates, the internal mesostructure introduced by the infill pattern can influence wave propagation, creating unique acoustic signatures for different infill densities.

To systematically evaluate localization performance across the plate surface, a grid of 25 impact points was defined. These points, denoted as $G_{k}$, where k=1,2,…,25, were spaced approximately 50 mm apart in both the x and y directions, covering the entire plate surface. This grid ensured comprehensive assessment of localization accuracy for impacts occurring both within and outside the sensor network boundaries.

To ensure consistent impact locations, the steel spheres were released through a vertical guide tube positioned above each designated grid point from a constant height of 540 mm, corresponding to an impact velocity of approximately 3.25 m/s. This setup constrained the balls’ trajectory during free fall. The AM plates were placed on unbonded foam blocks, providing a soft and non-restrictive support condition that approximates a free boundary while avoiding any mechanical constraints that could alter wave propagation. The spheres had material properties of $\rho =7830\;\mathrm{kg}/m^{3}$, and E=207GPa [42]. Three distinct impact energy levels were selected to evaluate the robustness of the localization algorithm across a range of excitation intensities. Three levels, denoted as $J_{a}$, $J_{b}$, and $J_{c}$, corresponded to steel ball diameters of 5.00 mm, 6.75 mm, and 10.00 mm, respectively.

**Figure 3:**
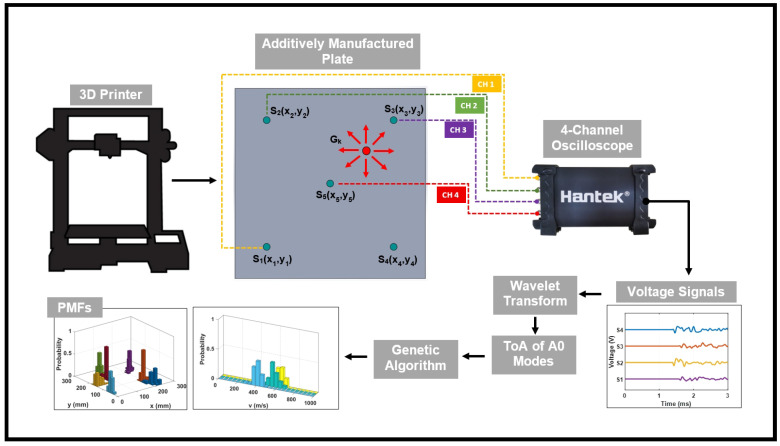
Schematic overview of the impact localization framework for AM plates with surface-mounted sensors to capture voltage signals generated by a low-velocity impact at source location $G_{k}$.

Table 2 summarizes the physical characteristics and corresponding impact energies for each impactor. The selection of three energy levels allows for the investigation of how impact magnitude influences signal amplitude, frequency content, and ultimately, localization accuracy. The positional uncertainty of the actual impact coordinates was maintained within ±5 mm. The experimental setup of the impact localization framework for AM plates with surface mounted sensors is shown in Figure 3. A four-channel Hantek oscilloscope (Qingdao Hantek Electronic Co., Ltd., Qingdao, Shandong, China) was used to capture transient voltage responses from the PZT sensors at a sampling rate of 1.25 MHz, recording 4096-sample waveforms per impact event. The sensors were connected directly to the oscilloscope’s high-impedance input channels via short, shielded BNC cables, with the oscilloscope set to DC-coupled, 1X probe mode. Temporal synchronization across all measurement channels was ensured through edge triggering, which initiated data acquisition when any sensor signal exceeded a fixed voltage threshold of 0.2 V. This threshold was selected to be more than three times greater than the maximum pre-arrival baseline noise amplitude observed in the raw data (0.0627 V), ensuring reliable triggering. The complete raw waveforms were saved for post-processing. The minimum signal-to-noise ratio across all experimental conditions was 27 dB.

This experimental design generated an extensive dataset encompassing multiple variables: three infill densities (30%, 50%, and 100%), two sensor networks ($N_{a}$ and $N_{b}$), and three impact energy levels ($J_{a}$, $J_{b}$, $J_{c}$). A total of 25 spatially distributed impact locations were defined on each plate. To assess repeatability, each location was impacted twice, resulting in 50 impacts per plate. This procedure was repeated for every plate tested. This comprehensive approach enabled systematic evaluation of the effects of each parameter on impact localization performance.

### 2.3. Wavelet-Based Feature Extraction and Genetic Algorithm Localization

Accurate impact localization requires the reliable extraction of wave arrival times from acquired sensor signals. The transient voltage responses from piezoelectric sensors were processed using the continuous wavelet transform (CWT) to obtain time–frequency representations. The CWT offers distinct advantages over conventional threshold-based methods, particularly for dispersive guided waves where frequency-dependent arrival times necessitate multi-resolution analysis. The CWT operation convolves the acquired signal V(t) with scaled and translated versions of a mother wavelet [43]:

(1) $$W_{\psi }\left(a,b\right)=\frac{1}{\sqrt{\left|a\right|}}\int_{-\infty }^{\infty }V\left(t\right),\psi^{*}\left(\frac{t-b}{a}\right)dt$$

where *a* represents the scale parameter, *b* denotes the translation parameter, and $\psi^{*}$ indicates the complex conjugate of the mother wavelet. A Morse wavelet was selected for its optimal time–frequency localization properties, enabling precise identification of arrival times across the frequency spectrum of interest [44].

Arrival time detection was implemented by analyzing wavelet coefficients within a predefined frequency band corresponding to the dominant guided wave modes. For each frequency component $f_{k}$ within the operational band $\left[f_{\min},f_{\max}\right]$, the arrival instant was identified as the earliest time at which the wavelet coefficient magnitude reached its maximum:

(2) $$\tau_{k}^{\mathrm{arr}}=\min\left\{t:\left|W_{\psi }\left(f_{k},t\right)\right|=\max_{\tau }\left|W_{\psi }\left(f_{k},\tau \right)\right|\right\}$$

This approach ensured consistent detection of the first wave arrival even in the presence of noise and reflections. The procedure generated time-of-arrival measurements for all five sensors, which were converted to relative time delays using sensor $S_{1}$ as a reference. The resulting three-element delay vector $\left[\Delta t_{21},\Delta t_{31},\Delta t_{41}\right]$ served as the input feature set for the optimization algorithm, enabling simultaneous estimation of impact coordinates and group velocity.

The inverse problem of determining impact location and wave velocity from measured time delays was solved using a genetic algorithm (GA). GAs are population-based optimization techniques that perform robust global searches without requiring gradient information or initial guesses, making them well suited for problems with complex error landscapes [45,46]. The algorithm minimizes the discrepancy between measured and theoretical time delays by evaluating candidate solutions against the wave propagation model [28]:

(3) $$L\left(X\right)=\sum_{i=2}^{4}{\left[\sqrt{{\left(x_{i}-x\right)}^{2}+{\left(y_{i}-y\right)}^{2}}-\sqrt{{\left(x_{1}-x\right)}^{2}+{\left(y_{1}-y\right)}^{2}}-v,\Delta t_{i1}\right]}^{2}$$

where the design variable vector $X={\left[x,\;y,\;v\right]}^{T}$ contains the unknown impact coordinates and the unknown wave velocity. The wave velocity *v* is treated as an unknown parameter to be estimated for each impact event, yielding an effective group velocity that best fits the measured time delays across all sensor pairs. The coordinates $x_{1},y_{1}$ represent the reference sensor $S_{1}$, while $\left(x_{i},y_{i}\right)$ represent the coordinates of the *i*-th sensor. The GA implementation employed a population of 50 individuals, stochastic uniform selection, intermediate crossover with an 80% rate, and elite preservation of the top 5% of solutions. The algorithm was run for 100 generations, with convergence typically achieved within 50–70 generations.

**Figure 4:**
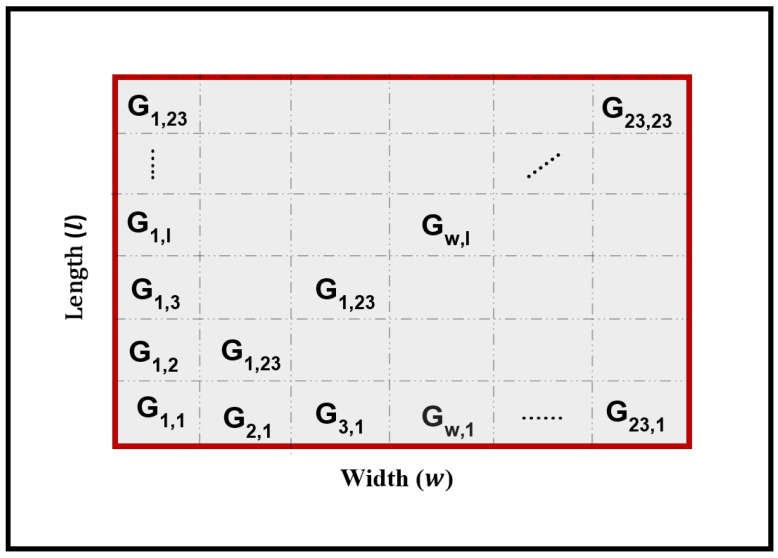
Spatial grid representation of the plate surface partitioned into a 23 × 23 matrix of cells, where each cell $G_{w,l}$ denotes a distinct spatial region for probabilistic impact mapping.

To quantify localization uncertainty and provide probabilistic interpretation of the results, the continuous solution space was discretized into a uniform grid. The plate surface, defined by constraints 0≤x≤l and 0≤y≤w, was discretized into a 23×23 matrix of cells, each denoted by $G_{w,l}$ where w=1,…,23 indexes the width direction and l=1,…,23 indexes the length direction, as illustrated in Figure 4. Each cell represents a distinct spatial region with dimensions of approximately 13 mm × 13 mm.

After GA convergence, the ensemble of candidate solutions from all populations and generations was mapped onto this grid. For each cell $G_{w,l}$, the count of solutions falling within its boundaries was recorded. The probability mass function (PMF) for impact coordinates was then computed as follows:

(4) $$P_{xy}\left(w,l\right)=\frac{n_{w,l}}{N_{\mathrm{total}}}$$

where $n_{w,l}$ is the number of solutions falling within cell $G_{w,l}$, and $N_{\mathrm{total}}$ is the total number of candidate solutions evaluated. It is important to note that $P_{xy}$ represents the spatial concentration of GA candidate solutions for a single impact event and is not a calibrated confidence level. It does not indicate the frequency with which the true impact falls into that cell across repeated experiments. Thus, the cell with the highest probability, denoted as $G_{w^{*},l^{*}}$, is interpreted as the most likely impact location:

(5) $$\left(w^{*},l^{*}\right)=\underset{w,l}{\mathrm{argmax}}\;P_{xy}\left(w,l\right)$$

This probabilistic approach provides several advantages over point estimates. The PMF reveals the spatial distribution of localization uncertainty, identifies multiple probable regions when signals are ambiguous, and enables visualization of confidence levels. Additionally, the same probabilistic framework was applied to the estimated group velocities, allowing for the characterization of velocity distributions for each impact event.

The discretization and probabilistic mapping allow GA outputs to be visualized, quantified, and systematically compared across experimental conditions. This structured framework integrates seamlessly with sensor layout considerations, providing a robust foundation for decision-making in structural health monitoring applications.

## 3. Results and Discussion

### 3.1. Time–Frequency Analysis of Impact-Induced Waves

**Figure 5:**
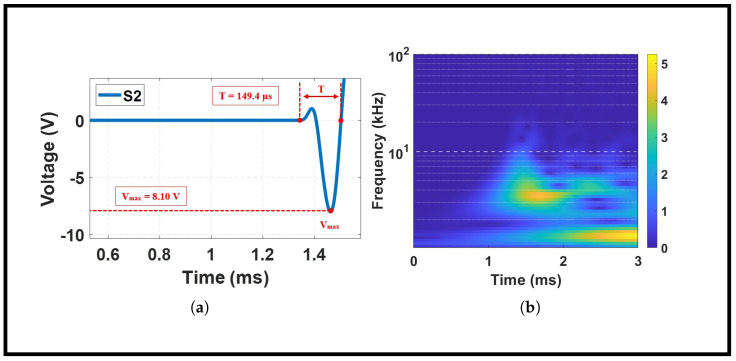
Time-domain signal and corresponding spectrogram for impact at $K_{23}$ on a 50% infill plate under $J_{c}$ using sensor network $N_{b}$. The color bar represents the amplitude of the continuous wavelet transform coefficients, with units in Volts (V).

Accurate impact localization depends critically on reliable identification of the first arrival wave mode from acquired sensor signals. Figure 5 presents a comprehensive view of the temporal and spectral characteristics of a representative impact event, serving as a pedagogical example for the waveform features observed throughout this study. Figure 5a shows the transient response recorded by sensor $S_{2}$ from a 50% infill plate subjected to a high-energy impact $J_{c}$ at location $K_{23}$ (*x* = 150 mm, *y* = 250 mm). The waveform exhibits a sharp negative voltage peak at the first arrival, reaching a maximum amplitude of −8.10 V, denoted as $V_{max}$. This initial peak corresponds to the compressive wavefront generated directly by the impact, propagating radially outward from the contact point. The prominence of this first arrival is essential for accurate time-of-arrival detection, as subsequent wave packets may be contaminated by reflections, mode conversions, or superimposed signals from multiple propagation paths. The time duration of the first complete oscillation cycle, measured from signal onset to the completion of the first positive–negative excursion, is *T* = 149.4 μs. This temporal parameter provides insight into the dominant frequency content of the initial wave packet and serves as a consistency check for arrival time detection algorithms.

Figure 5b complements the time-domain analysis with a spectrogram displaying the signal’s frequency content evolution over time. The frequency axis is logarithmic, spanning from 1 to 100 kHz, with color intensity indicating spectral amplitude. Several important observations emerge from this representation. First, the dominant energy is concentrated below 10 kHz, with the peak intensity occurring between 1 and 2 ms, accurately aligned with the first arrival cycle identified in Figure 5a. Second, the energy distribution exhibits a characteristic decay pattern, with higher frequencies attenuating more rapidly than lower frequencies. This low-frequency dominance suggests that the impact primarily excites the fundamental antisymmetric ($A_{0}$) Lamb wave mode, which is known to be sensitive to material properties, thickness variations, and internal structure. It should be mentioned that low-velocity impacts in thin plate structures are widely reported to excite predominantly the fundamental antisymmetric ($A_{0}$) Lamb mode, with minimal contribution from the ($S_{0}$) mode at low frequency–thickness products. Prior studies show through dispersion-curve analysis that the $A_{0}$ mode dominates wave propagation below 50–100 kHz-mm, which corresponds to the frequency range observed in our measurements [28].

The waveform characteristics observed in Figure 5 establish a baseline for interpreting the effects of infill density presented in subsequent sections. Variations in peak amplitude, first-cycle duration, and spectral content across different infill densities and impact energies provide quantitative metrics for understanding how internal mesostructure influences wave propagation and, consequently, localization accuracy.

### 3.2. Influence of Infill Density on Waveform Characteristics

**Figure 6:**
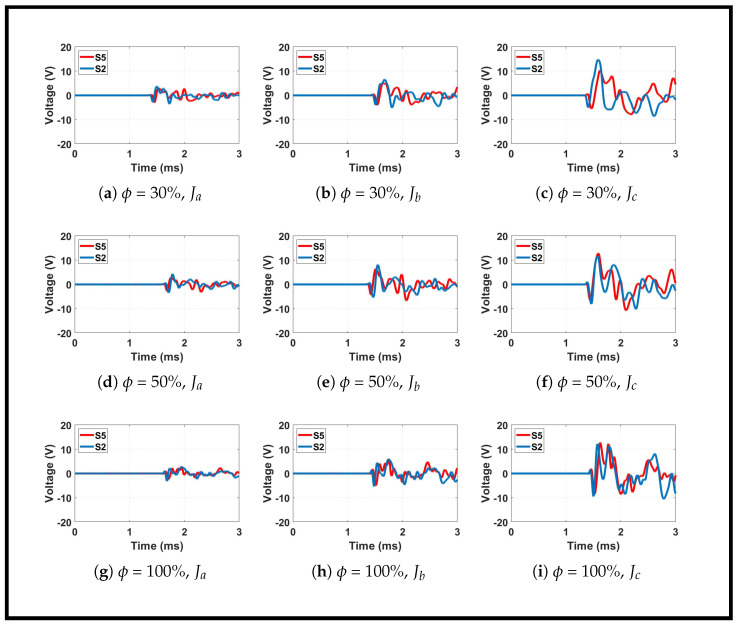
Waveform signals from sensors $S_{2}$ and $S_{5}$ for impacts at location $K_{23}$ across three infill densities and three impact energy levels using sensor network $N_{a}$.

**Figure 7:**
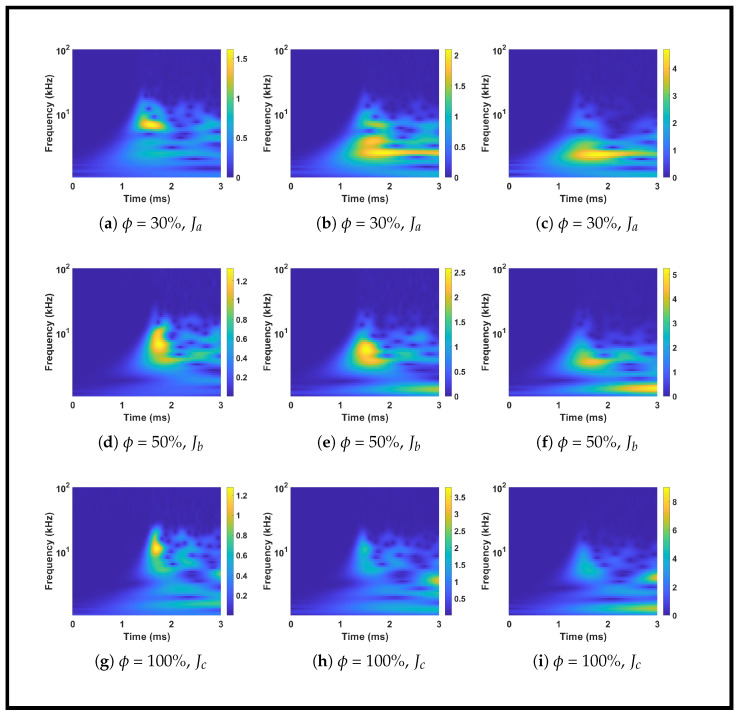
Frequency spectra of signals from sensor $S_{2}$ for impacts at location $K_{23}$ across three infill densities and three impact energy levels using sensor network $N_{a}$. The color bar represents the amplitude of the continuous wavelet transform coefficients, with units in Volts (V).

The influence of infill density on impact-generated wave propagation was examined through time-domain waveforms, frequency spectra, and quantitative metrics. Figure 6 presents the waveform signals captured from sensors $S_{2}$ and $S_{5}$ for impacts at location $K_{23}$ (x = 150 mm, y = 250 mm) across three infill densities (30%, 50%, and 100%) and three impact energy levels ($J_{a}$, $J_{b}$, $J_{c}$). Figure 7 shows the corresponding frequency spectra for sensor $S_{2}$, revealing systematic shifts in spectral content with infill density. Table 3 quantifies the maximum voltage ($V_{max}$) of the first arrival cycle and the voltage ratios across different impact energies, providing a numerical basis for the observed trends.

#### 3.2.1. Time-Domain Waveform Analysis

Figure 6 reveals several important characteristics of wave propagation in AM plates with varying infill density. First, sensors $S_{2}$ and $S_{5}$ are positioned at nearly equal distances from the impact location $K_{23}$. Despite this near-equidistant configuration, the waveforms exhibit minor variations in first arrival timing. Sensor $S_{2}$ shows a slightly earlier arrival than $S_{5}$, with the time difference less than 40 μs depending on infill density and impact energy. This subtle disparity can be attributed to the impact positioning relative to the sensor network geometry. While nominally equidistant, the actual path lengths differ slightly, and the wave propagation paths encounter different local material configurations within the heterogeneous infill structure.

Second, the first arrival waveforms from both sensors display similar shapes and magnitudes for a given infill density and impact energy. The initial negative peaks align closely in amplitude and duration, indicating that the direct compressive wavefront experiences comparable propagation conditions along the initial segments of both paths. However, beyond the first arrival cycle, the waveforms diverge significantly. This divergence becomes particularly evident in the later portions of the signals, where reflections, mode conversions, and scattered waves produce distinct signatures at each sensor. The internal mesostructure of the AM plates creates spatially varying propagation paths. Waves traveling to different sensors encounter different arrangements of infill struts, voids, and material interfaces, leading to unique signal modifications after the first arrival. The first arrival itself remains relatively unaffected because it propagates through the most direct path, but subsequent wave interactions with the internal structure differ substantially between sensor locations.

Third, increasing the infill density systematically alters the waveform characteristics. The 30% infill plate produces relatively clean signals with minimal high-frequency oscillations. The 50% infill plate exhibits higher peak amplitudes, particularly evident in the $J_{b}$ and $J_{c}$ impacts, where $V_{max}$ reaches 5.30 V and 8.10 V, respectively. The 100% infill plate shows the most complex waveforms, with pronounced high-frequency oscillations superimposed on the fundamental wave packet. This reflects stronger and more continuous propagation paths as the infill density increases. Higher density provides more material continuity for wave transmission, enabling larger amplitude signals while simultaneously introducing greater complexity through multiple scattering events within the denser internal structure.

#### 3.2.2. Spectral Analysis and Frequency Content

Figure 7 illustrates the systematic shift in frequency content with infill density. The 30% and 50% infill plates consistently exhibit spectral energy concentrated below 10 kHz, with amplitudes decaying rapidly above this threshold. In contrast, the 100% infill plate shows significant spectral components extending well beyond 10 kHz, reaching up to 30–40 kHz in some cases. This spectral broadening in denser plates arises directly from their increased stiffness and continuous internal architecture, which support higher-order wave modes and faster vibrations. The porous and compliant structure of the 30% infill plate acts as an effective mechanical low-pass filter, attenuating high-frequency components that would otherwise complicate the signal. Consequently, higher-infill plates produce more complex signal signatures with pronounced high-frequency content.

The internal mesostructure of the additively manufactured plates is governed by the cubic infill pattern, which creates a periodic lattice of interconnected struts and voids. These mesostructural parameters fundamentally govern wave propagation through the plates. The periodic arrangement of struts and voids acts as a scattering medium for elastic waves, with the scattering cross-section scaling with the ratio of pore size to wavelength. As the infill density decreases, the pore size increases, enhancing wave scattering and attenuation at higher frequencies. This explains the low-pass filtering behavior observed for the 30% infill plate, where high-frequency components are selectively attenuated. Conversely, the 100% infill plate, with minimal pore size and maximal material continuity, supports broadband wave propagation with minimal scattering loss. The relationship between porosity and wave velocity is also well established [47,48].

An additional trend observed in Figure 7 is the downward shift in the dominant frequency of the first arrival as the impact magnitude increases. For all infill densities, the spectral peak shifts toward lower frequencies when progressing from $J_{a}$ to $J_{c}$. This behavior aligns with the principles of contact mechanics, in which larger impactors with greater mass and contact area produce longer contact durations, thus exciting lower-frequency forcing functions. The effect is most pronounced in the 30% infill plate, where the naturally filtered response accentuates this frequency shift.

**Figure 8:**
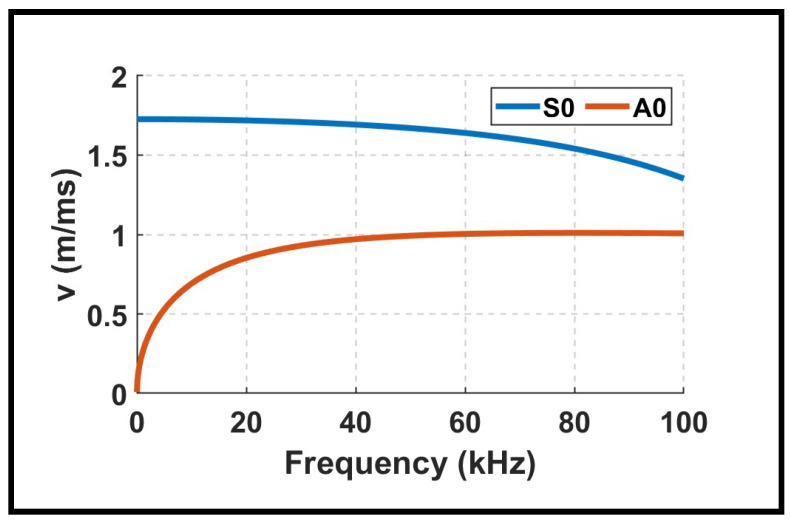
Group velocity curves of fundamental Lamb modes for a solid PLA plate with a 5.3 mm thickness.

To confirm the Lamb wave mode excited by low-velocity impacts, theoretical dispersion curves, as shown in Figure 8, were computed for a solid PLA plate with a thickness of 5.3mm, using material properties of $\rho =1.190\;g/{\mathrm{cm}}^{3}$, E=3.16GPa, and ν=0.33. At the dominant frequency of f≈10kHz observed in the spectrograms, the fundamental antisymmetric mode A0 has a group velocity of approximately 690m/s, while the symmetric mode S0 propagates at about 1724m/s. The experimentally estimated group velocities for the 100% infill plate, averaging at about 550 from m/s, are in good agreement with the theoretical A0 velocity, confirming that the impact primarily excites the A0 mode. The small deviation can be attributed to the inherent mesostructure of additively manufactured plates, where even fully dense specimens do not behave exactly as bulk solids due to layer-wise fabrication and microscopic voids. Overall, the effective bandwidth of the signals supports the conclusion that the A0 mode is the primary propagating mode under the tested conditions.

#### 3.2.3. Quantitative Voltage Analysis

Table 3 quantifies the maximum voltage of the first arrival cycle for all experimental conditions. Several important observations emerge from these data. For identical impact energy, sensor voltage does not vary monotonically with infill density. Under $J_{a}$ impact, the 50% infill plate produces the highest $V_{max}$ (3.40 V), followed by 100% (3.10 V) and 30% (2.76 V). The 100% plate does not yield the highest peak voltage because its energy distributes across a wider frequency band, reducing the concentration of energy in the first arrival cycle. The 50% infill plate represents an optimal compromise with sufficient structural continuity to transmit strong signals, yet enough filtering to concentrate energy in the fundamental mode.

Additionally, the voltage ratio between successive impact levels shows a clear increasing trend with infill density. For the 30% infill plate, the average voltage ratio across impact levels is approximately 1.35. This increases to 1.54 for the 50% infill plate and reaches 1.75 for the 100% infill plate. This progressive increase indicates that denser structures respond more sensitively to increasing impact energy.

Furthermore, the voltage increase is consistently lower than the corresponding impact energy ratio from Table 2. For example, the energy ratio between $J_{b}$ and $J_{a}$ is approximately 2.53, yet the voltage ratios range from 1.42 (30% infill) to 1.71 (100% infill). This discrepancy arises from contact mechanics in which larger diameter impactors distribute force over larger contact areas and longer durations, producing a different excitation spectrum than simply scaling impact energy would suggest. The voltage ratio thus represents a complex convolution of impact energy, contact mechanics, and material-dependent wave propagation characteristics.

#### 3.2.4. Synthesis

Collectively, the time-domain, spectral, and quantitative analyses reveal that infill density fundamentally governs wave propagation in AM plates. Lower densities (30%) act as mechanical filters, producing clean, low-frequency signals that are ideal for arrival time detection, but with reduced amplitude. Higher densities (100%) support complex, broadband propagation with larger overall energy but greater signal complexity. The intermediate 50% density offers a compromise, delivering the highest peak amplitudes while maintaining reasonably clean waveforms.

### 3.3. Impact Localization Performance Across the Plate Surface

**Figure 9:**
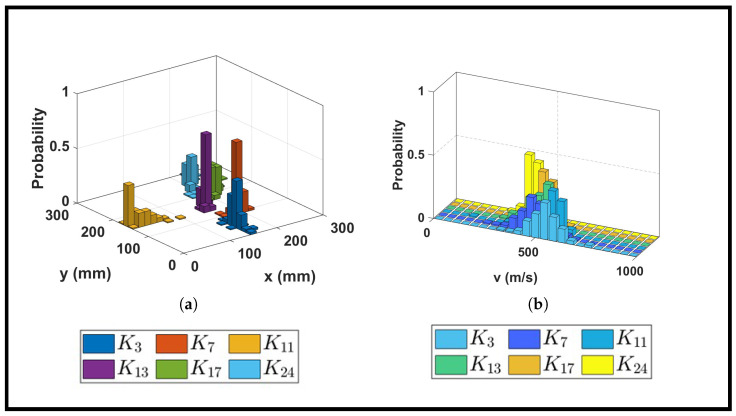
Probability distributions for six impact locations on 30% infill plate using sensor network $N_{b}$: (**a**) spatial probability mass functions $P_{xy}$; (**b**) corresponding group velocity distributions $P_{v}$.

While the previous section examined waveform characteristics at a fixed impact location ($K_{23}$) across varying infill densities, this section evaluates the genetic algorithm’s localization performance at multiple impact points distributed across the plate surface. For this analysis, the 30% infill plate and sensor network $N_{b}$ were selected to establish the baseline performance. Figure 9 presents the probability distributions of impact coordinates $P_{xy}$ and corresponding group velocity distributions $P_{v}$ for six representative impact locations. Table 4 provides a quantitative summary of the predicted coordinates, uncertainties, group velocities, and normalized errors for each location. The prediction error in Table 4 was calculated as the difference between the actual and predicted impact coordinates. The percentage error was obtained by dividing by the characteristic length of the respective plate to normalize predictions. The mean absolute percentage error and uncertainty (standard deviation) for *x*- and *y*-coordinates were calculated as follows:

(6) $$\mu =\frac{1}{N}\sum_{i=1}^{N}\left|e_{i}\right|$$

(7) $$\sigma =\sqrt{\frac{1}{N}\sum_{i=1}^{N}\left(\right|e_{i}{\left|-\mu \right)}^{2}}$$

where $e_{i}$ is the prediction error for the *i*-th sample and *N* is the total number of GA runs. Figure 9a shows the probability mass functions (PMFs) of predicted impact coordinates for six locations across the 30% infill plate. The PMFs reveal tightly clustered probability distributions around each true impact point, demonstrating the GA’s ability to consistently converge to the correct spatial region despite the sparse internal structure of the plate.

Among all impact points, $K_{3}$ exhibits localization accuracy with less than 1% error in both coordinates and a maximum spatial probability of 43%. Impact points $K_{7}$ and $K_{13}$ demonstrate even stronger convergence, with maximum probabilities exceeding 65% and spatial uncertainties below ±7 mm in both coordinate directions. The normalized errors for these locations remain below 4%. The high probability at $K_{13}$ (68%) suggests favorable sensor geometry relative to this impact point.

Impact points $K_{17}$ and $K_{24}$ show intermediate performance, with maximum probabilities of 27% and 29%, respectively, and errors below 5% in both coordinates. While these probabilities are lower than those at $K_{7}$ and $K_{13}$, the spatial distributions remain concentrated around the true locations. The largest localization error occurs at impact point $K_{11}$. This elevated error, particularly in the x-direction (9%), can be attributed to the geometric configuration of sensors relative to this impact point. $K_{11}$ lies in a region where multiple sensors are nearly equidistant, creating near-singular conditions in the time-difference equations. Small variations in arrival time estimates due to noise or wave dispersion are amplified in such geometries, leading to greater solution variability. Despite this challenge, the y-coordinate error remains low (2%), indicating that the GA still captures the dominant propagation direction accurately.

Figure 9b presents the probability distributions of estimated group velocities for the same six impact locations. The distributions exhibit predominantly normal shapes, with mean velocities ranging from approximately 386 m/s at $K_{24}$ to 543 m/s at $K_{3}$, as summarized in Table 4. Notably, the velocity distribution for $K_{24}$ displays a right-skewed character, with maximum probability concentrated at 48% and relatively low uncertainty (±26 m/s). The normalized errors for this location remain below 3% in both coordinates, despite the non-Gaussian velocity distribution. This right skew suggests that waves propagating from $K_{24}$ to the sensor network experience a range of effective propagating paths due to alignment with stiffer directions in the cubic infill architecture.

Additionally, the variation in estimated velocity across different impact locations on the same plate is relatively significant. The 30% infill structure is not a homogeneous medium, because it consists of periodic cubic cells with alternating material and void regions. Waves propagating in different directions encounter different effective stiffnesses depending on their alignment with the infill struts. Additionally, local variations in print quality, slight thickness variations, and boundary interactions contribute to location-dependent effective wave speeds. The results in Figure 9 and Table 4 demonstrate that impact localization accuracy in AM plates depends not only on infill density but also on the specific impact location relative to the sensor network. These findings establish that the GA-based approach combined with probabilistic mapping provides reliable impact localization across the surface of the AM plate.

### 3.4. Effect of Infill Density on Impact Localization

**Figure 10:**
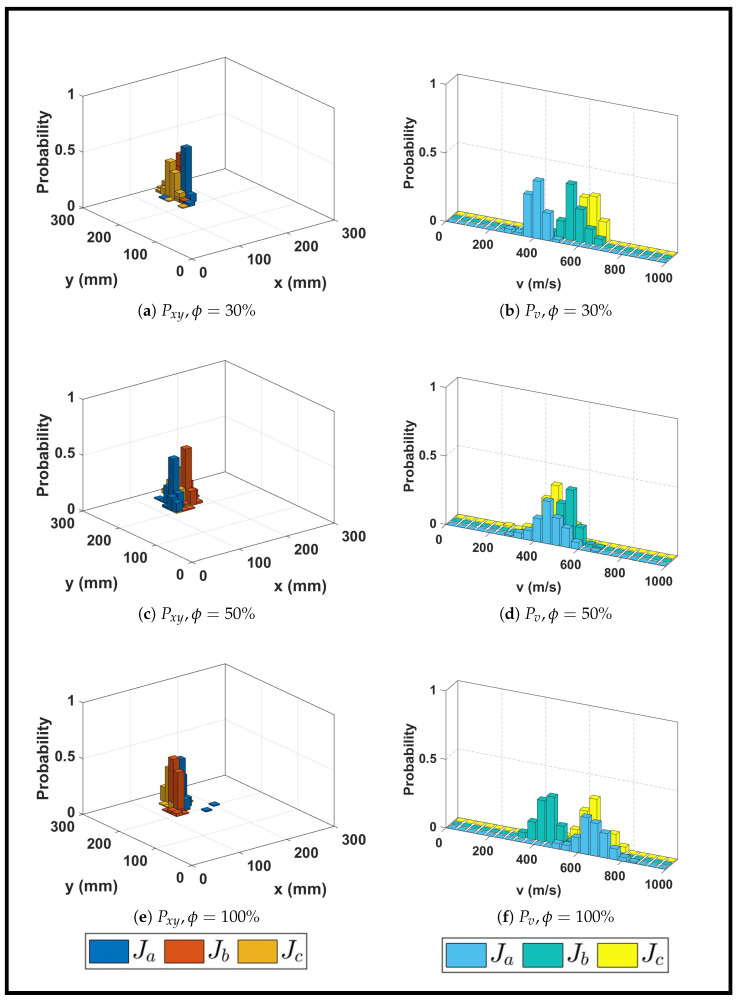
Probability distributions for impacts at location $K_{23}$ across three infill densities and three impact energy levels using sensor network $N_{a}$.

While the previous section examined localization performance across multiple impact locations on a single infill density (30%), this section investigates how infill density itself influences localization accuracy and wave propagation characteristics. Figure 10 presents the probability distributions of impact coordinates $P_{xy}$ and group velocity $P_{v}$ for impacts at a fixed location $K_{23}$ (*x* = 150 mm, *y* = 250 mm) across three infill densities and three impact energy levels. Table 5 provides a quantitative summary of the predicted coordinates, uncertainties, group velocities, maximum probabilities, and normalized errors for all nine experimental conditions.

#### 3.4.1. Spatial Localization Accuracy

For the 30% infill plate, the spatial probability mass functions in Figure 10a show well-defined clusters around the true impact location $G_{12,19}$ across all three impact energies. The maximum spatial probabilities $P_{xy}$ range from 32% to 47%, with the mean predicted coordinates deviating by less than 4% in the x-direction and less than 6% in the y-direction from the true location. The candidate grid cells identified by the GA are all immediately adjacent to the true impact cell, demonstrating consistent convergence to the correct region despite the sparse internal structure. Averaged across all impact energies, the normalized localization errors remain below 3% in both coordinate directions.

The 50% infill plate exhibits spatial localization performance broadly similar to that of the 30% case. The maximum spatial probabilities range from 31% to 49%, and the mean coordinate estimates show marginally larger deviations. Despite this increased spread, the predicted grid cells remain adjacent to the true impact cell $G_{12,19}$. When averaged across all impact magnitudes, the normalized localization errors remain below 5%, demonstrating that the algorithm maintains reliable performance even as the internal structure becomes stiffer and more continuous. The 50% plate achieves the highest peak probabilities among all densities for some impact levels, suggesting that its intermediate structure may provide an optimal balance between signal clarity and amplitude.

For the 100% infill plate, the spatial probability distributions remain strongly concentrated around the true impact region, with the maximum probabilities reaching up to 46%. Minor deviations appear primarily in the $J_{a}$ case, where a small subset of solutions shifts slightly away from the dominant cluster but the overall distribution remains tightly concentrated. The mean predicted coordinates for all impacts remain close to the true location, with normalized localization errors averaging below approximately 4% across all impact magnitudes.

Interestingly, the effect of infill density on localization accuracy is relatively modest. Across all three densities, the mean localization errors remain below 6%, with most conditions achieving errors below 4%. This indicates that while signal characteristics vary significantly with infill density (as shown in Figure 6 and Figure 7), the GA-based approach remains robust across the entire range of infill densities studied.

#### 3.4.2. Group Velocity Dependence on Infill Density and Impact Energy

The group velocity distributions in Figure 10b and Table 5 reveal systematic and physically meaningful trends. For the 30% infill plate, the mean velocities increase monotonically with the impact energy. This progression reflects the broader frequency content excited by higher-energy impacts. The GA’s estimated velocity represents the dominant wave packet, which shifts toward higher values as higher-energy impacts excite higher-frequency components that propagate faster in the $A_{0}$ mode.

The 50% infill plate exhibits velocity distributions with relatively narrow and symmetric shapes. The mean velocities show less variation with impact energy compared to the 30% case, suggesting that the 50% infill structure provides more consistent wave propagation characteristics across excitation frequencies.

The 100% infill plate reaches the highest velocities, with an average mean value of 640 m/s, and exceeding 800 m/s for several GA solutions. The velocity distributions exhibit approximately normal shapes, with peaks corresponding to each impact level with greater width than the 50% case, indicating more variability due to the complex wave propagation in the solid structure.

The observed increase in group velocity with infill density can be explained through classical wave propagation theory. In guided waves, the effective group velocity scales approximately with the square root of the stiffness-to-density ratio: $v\propto \sqrt{E/\rho }$. As the infill density increases, the printed structure contains more continuous load-bearing paths and fewer voids. This elevates the effective stiffness *E* more rapidly than the mass density ρ, increasing the ratio E/ρ and, consequently, the group velocity. Despite these sources of uncertainty, the GA demonstrates strong and consistent performance across all impact magnitudes and infill densities, with mean localization errors below 6% for all conditions.

### 3.5. Influence of Infill Density on Computational Efficiency

**Figure 11:**
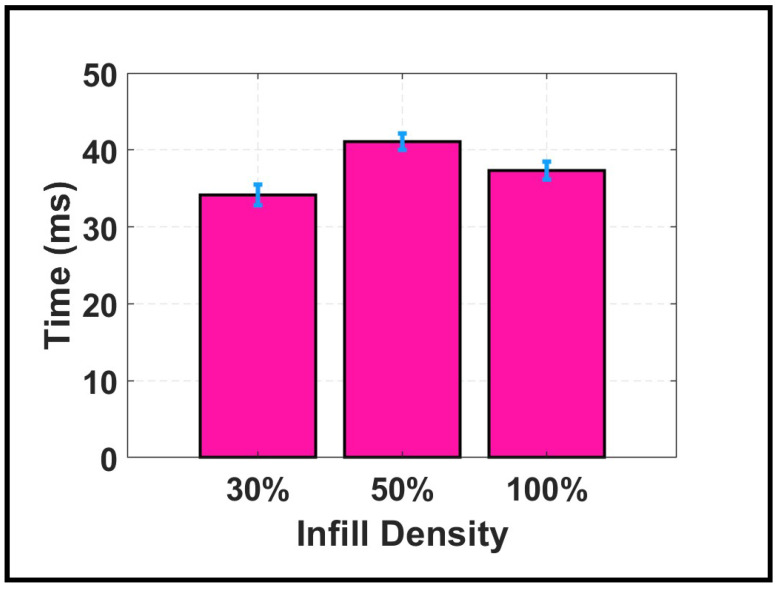
Average computational time for impact localization across three infill densities based on 500 independent GA runs per configuration. Error bars represent standard deviations.

Computational efficiency is an important consideration for practical SHM deployment. Figure 11 presents the average computation time required for the GA to localize impacts and estimate wave velocity for each infill density based on 500 independent runs per impact. All calculations were performed on an 11th Generation Intel^©^ Core i7 processor using MATLAB^©^ (Version R2024a). The reported times represent the statistical summary of 500 independent GA runs per plate configuration. The processing time is the time required for the GA framework to localize impacts and estimate the wave speed of propagation waves.

The recorded average computation times in Figure 11 show distinct but relatively close values across infill densities: 34.15±1.37 ms for the 30% infill plate, 41.08±1.09 ms for the 50% infill plate, and 37.31±1.18 ms for the 100% infill plate. The low standard deviations across all conditions indicate stable and repeatable algorithm performance, with computational cost exhibiting minimal sensitivity to infill density. The relatively weak correlation between computational cost and infill density can be understood by considering two competing factors: signal resolution and signal amplitude. Clean signals such as those obtained from the 30% infill plate minimized the search space complexity and, consequently, enabled rapid convergence. High-amplitude signals from the 50% and 100% infill plates provided strong initial cues that guided the search, partially compensating for the increased waveform complexity.

Overall, the influence of infill density on computational cost is modest. The maximum observed difference between any two densities was less than 20% of the minimum time, indicating that the GA-based approach maintains reasonable efficiency across the entire range of infill configurations studied herein.

These findings have practical implications for SHM system design. The additional computational burden associated with higher infill densities may be acceptable for post-event assessment but could pose challenges for real-time applications requiring immediate damage assessment. In such cases, the 30% infill configuration offers not only superior localization accuracy but also the lowest computational demand.

## 4. Conclusions

To investigate the effect of infill density on impact localization in additively manufactured structures, 3D-printed PLA plates with three infill densities and integrated piezoelectric sensors were fabricated and tested. Under systematic experiments, these plates were subjected to three impact energy levels at multiple impact locations across the plate surface, using two different sensor network configurations. The acquired signals were processed using continuous wavelet transform and a genetic algorithm framework to simultaneously estimate impact coordinates and group velocity. The key findings are summarized below:

- The lower infill structures act as mechanical low-pass filters, producing clean and low-frequency signals. In contrast, higher infill densities support complex and broadband wave propagation with higher overall energy.
- The voltage ratios of first arrival increase systematically with infill density. However, the dominant frequency of the first arrival in all infill densities shifts toward lower frequencies when increasing the impact energy level. This behavior aligns with contact mechanics principles, in which larger impactors with greater mass and contact area produce a longer contact duration.
- Group velocity increases with both impact energy level and infill density. This trend follows classical wave theory, as increased infill density elevates effective stiffness more rapidly than mass density.
- Group velocity varies with impact location on the same plate, attributed to the heterogeneous nature of AM structures with periodic voids and material regions.
- The genetic algorithm integrated with wavelet-based feature extraction demonstrated robust performance across all experimental conditions, simultaneously estimating impact coordinates and group velocity with an average error of less than 6%.
- Spatial probability mass functions revealed tightly clustered predictions around the true impact locations, with maximum probabilities reaching 68% and uncertainties below ±15 mm.
- Computational efficiency varies modestly with infill density, with the 30% configuration offering the highest accuracy with the lowest computational demand.

This work establishes quantitative relationships among infill density, wave propagation, and localization performance in additively manufactured structures. Despite the robustness of the genetic algorithm in estimating impact coordinates, it must be acknowledged as a limitation that utilizing an effective group velocity for each event is a simplification of the complex, path-dependent wave propagation in highly anisotropic AM structures. While this approach successfully eliminates the need for a priori material data, future work could explore direction-dependent velocity models to further refine localization accuracy in architectures with extreme heterogeneity.

Future work may also extend this framework to alternative infill patterns, anisotropic architectures, and real-time implementation on resource-constrained hardware. Hybrid machine learning approaches to further enhance impact localization capabilities would also advance the practical deployment of self-sensing AM structures.

## Figures and Tables

**Table 1:** Physical and geometric properties of the additively manufactured PLA plates with different infill densities.

Infill	Mass (g)	Thickness (mm)	Width (mm)	Length (mm)	Volume (cm^3^)	Density (g/cm^3^)
30 %	270 ± 2.6	5.3 ± 0.11	294 ± 0.91	294 ± 0.92	458.11	0.589
50 %	369 ± 1.5	5.4 ± 0.08	296 ± 0.62	296 ± 0.63	473.13	0.780
100 %	558 ± 1.3	5.5 ± 0.09	292 ± 0.57	292 ± 0.53	468.95	1.190

**Table 2:** Physical characteristics of the three steel impactors used in the experimental tests, including their diameters, masses, and corresponding impact energies.

Impact	Diameter (mm)	Mass (g)	*U* (mJ)	*U* Ratio
$J_{a}$	5.00	0.513	1.15	—
$J_{b}$	6.75	1.260	2.91	2.53
$J_{c}$	10.00	4.110	10.03	3.45

**Table 3:** Characteristics of the first arrival cycle from sensor $S_{2}$ for impacts at location $K_{23}$ across three infill densities and three impact energy levels using sensor network $N_{a}$.

Infill	Impact	$V_{\max}$ (V)	$V_{\max}$ Ratio	T (μs)	*f* (Hz)
30%	$J_{a}$	2.76	—	100.8	9920.6
30%	$J_{b}$	3.92	1.42	121.6	8223.7
30%	$J_{c}$	5.00	1.28	124.8	8012.8
50%	$J_{a}$	3.40	—	130.4	7668.7
50%	$J_{b}$	5.30	1.56	140.8	7102.3
50%	$J_{c}$	8.10	1.52	149.4	6693.4
100%	$J_{a}$	3.10	—	115.2	8680.6
100%	$J_{b}$	5.30	1.71	124.0	8064.5
100%	$J_{c}$	9.42	1.78	146.4	6830.6

**Table 4:** Localization performance for six impact locations on 30% infill plate using sensor network $N_{b}$.

Infill	Network	*K*	$P_{\mathrm{xy}}$	$\bar{x}\pm \sigma_{x}$ mm	$\bar{y}\pm \sigma_{y}$ mm	$P_{v}$	$\bar{v}\pm \sigma_{v}$ m/s	$E_{x}$	$E_{y}$
30%	$N_{b}$	$K_{3}$	43%	152.2±2.7	47.4±13.7	29%	543.3±73.6	0.75%	0.88%
30%	$N_{b}$	$K_{7}$	66%	191.2±7.7	98.6±3.6	28%	469.3±75.0	2.99%	0.48%
30%	$N_{b}$	$K_{11}$	38%	21.6±19.2	156.1±4.1	33%	527.0±71.4	9.65%	2.07%
30%	$N_{b}$	$K_{13}$	68%	160.7±3.5	151.6±6.3	34%	460.1±58.7	3.64%	0.54%
30%	$N_{b}$	$K_{17}$	27%	212.9±9.7	195.3±10.1	40%	474.9±37.3	4.39%	1.60%
30%	$N_{b}$	$K_{24}$	29%	206.9±10.2	251.0±15.6	48%	385.9±26.1	2.35%	0.34%

**Table 5:** Localization performance for impacts at $K_{23}$ across three infill densities and three impact energy levels using sensor network $N_{a}$.

Infill	Impact	$P_{\mathrm{xy}}$	$G_{w,l}$	$\bar{x}\pm \sigma_{x}$ mm	$\bar{y}\pm \sigma_{y}$ mm	$P_{v}$	*v*-Range m/s	$\bar{v}\pm \sigma_{v}$ m/s	$E_{x}$	$E_{y}$
30%	$J_{a}$	47%	$G_{13,18}$	161.0±4.7	233.2±11.4	42%	391–434	402±40	3.67%	5.60%
30%	$J_{b}$	39%	$G_{13,19}$	162.0±4.4	250.3±15.8	41%	521–565	559±52	4.00%	0.10%
30%	$J_{c}$	32%	$G_{12,20}$	154.6±3.7	254.1±15.1	32%	608–652	600±53	1.53%	1.37%
50%	$J_{a}$	43%	$G_{11,18}$	133.7±4.3	224.4±11.9	31%	434–478	466±66	5.43%	8.53%
50%	$J_{b}$	49%	$G_{13,18}$	166.9±6.6	228.6±9.1	39%	521–565	519±55	5.63%	7.13%
50%	$J_{c}$	31%	$G_{12,19}$	156.5±6.1	246.7±14.8	37%	434–478	452±54	2.17%	1.10%
100%	$J_{a}$	44%	$G_{12,18}$	155.1±7.9	229.7±9.5	27%	608–652	660±68	1.70%	6.77%
100%	$J_{b}$	46%	$G_{11,18}$	138.2±5.0	223.1±8.3	34%	434–478	439±50	3.93%	8.97%
100%	$J_{c}$	36%	$G_{12,19}$	149.7±4.9	248.5±11.8	35%	608–652	621±61	0.10%	0.50%

## Data Availability

Data are available upon request.

## Nomenclature

*Roman Symbols*	
*a*	Scale parameter in the continuous wavelet transform (CWT)
*b*	Translation parameter in the CWT
$d_{31}$	Piezoelectric strain coefficient of PZT sensors (C/N)
*E*	Young’s modulus (GPa)
$e_{i}$	Prediction error for the *i*-th GA population member (mm)
*f*	Frequency (kHz)
$G_{w,l}$	Spatial grid cell indexed by width *w* and length *l*
*N*	Number of GA runs or samples
$P_{xy}$	Probability of predicted impact coordinates falling in cell (x,y)
$P_{v}$	Probability of predicted group velocity
*T*	Period of first wave cycle (μs)
*U*	Impact energy (mJ)
V(t)	Measured voltage signal (V)
$V_{\max}$	Maximum voltage amplitude of first arrival (V)
*v*	Estimated group velocity of guided waves (m/s)
x,y	Impact coordinates in plate reference frame (mm)
$\bar{x},\bar{y}$	Mean predicted coordinates (mm)
*Greek Symbols*	
Δt	Time delay between sensor arrivals (μs)
μ	Mean absolute percentage error (%)
ρ	Density (g/cm^3^ or kg/m^3^)
σ	Standard deviation
$\tau_{k}$	Arrival time of direct wave packet at sensor *k* (μs)
ϕ	Infill density of AM plates (%)
ψ, $\psi^{*}$	Mother wavelet and its complex conjugate
*Subscripts and Superscripts*	
*i*	Sensor or sample index
*k*	Frequency bin or sensor index
max	Maximum value
min	Minimum value
∗	Optimal value (e.g., $G_{w^{*},l^{*}}$)
arr	Arrival (e.g., $\tau_{k}^{\mathrm{arr}}$)
*Abbreviations*	
S0	Fundamental symmetric Lamb wave mode
A0	Fundamental antisymmetric Lamb wave mode
AM	Additive manufacturing
CWT	Continuous wavelet transform
FDM	Fused deposition modeling
GA	Genetic algorithm
PLA	Polylactic acid
PMF	Probability mass function
PZT	Lead zirconate titanate piezoelectric sensor
SHM	Structural health monitoring
TDoA	Time difference of arrival
ToA	Time of arrival
